# Backmasking in the yeast genome: encoding overlapping information for protein-coding and RNA degradation

**DOI:** 10.1093/nar/gkw683

**Published:** 2016-08-04

**Authors:** S. Aylin Cakiroglu, Judith B. Zaugg, Nicholas M. Luscombe

**Affiliations:** 1 The Francis Crick Institute, 44 Lincoln's Inn Fields Laboratory, London WC2A 3LY, UK; 2 European Molecular Biology Laboratory, 69117 Heidelberg, Germany; 3 UCL Genetics Institute, University College London, London WC1E 6BT, UK; 4 Okinawa Institute of Science & Technology Graduate University, Okinawa 904-0495, Japan

## Abstract

Backmasking is a recording technique used to hide a sound or message in a music track in reverse, meaning that it is only audible when the record is played backwards. Analogously, the compact yeast genome encodes for diverse sources of information such as overlapping coding and non-coding transcripts, and protein-binding sites on the two complementary DNA strands. Examples are the consensus binding site sequences of the RNA-binding proteins Nrd1 and Nab3 that target non-coding transcripts for degradation. Here, by examining the overlap of stable (SUTs, stable unannotated transcripts) and unstable (CUTs, cryptic unstable transcripts) transcripts with protein-coding genes, we show that the predicted Nrd1 and Nab3-binding site sequences occur at differing frequencies. They are always depleted in the sense direction of protein-coding genes, thus avoiding degradation of the transcript. However in the antisense direction, predicted binding sites occur at high frequencies in genes with overlapping unstable ncRNAs (CUTs), so limiting the availability of non-functional transcripts. In contrast they are depleted in genes with overlapping stable ncRNAs (SUTs), presumably to avoid degrading the non-coding transcript. The protein-coding genes maintain similar amino-acid contents, but they display distinct codon usages so that Nrd1 and Nab3-binding sites can arise at differing frequencies in antisense depending on the overlapping transcript type. Our study demonstrates how yeast has evolved to encode multiple layers of information—protein-coding genes in one strand and the relative chance of degrading antisense RNA in the other strand—in the same regions of a compact genome.

## BACKGROUND

About 85% of the *Saccharomyces cerevisiae* genome is transcribed (1): in addition to messenger RNAs (mRNA) and the classical non-coding RNAs (ncRNA) such as small nuclear RNAs, transfer RNAs and ribosomal RNAs (2), other ncRNAs with unknown functions have been described (3,4). Some of these latter RNAs play a role in gene regulation (5–12) and they have been classified as stable uncharacterised transcripts (SUTs) and cryptic unstable transcripts (CUTs), depending on whether or not their expression is observed in wild-type cells (SUTs) or only upon deletion of RRP6, a component of the exosome (CUTs) (9).

Transcription of mRNAs and most SUTs is terminated by a pathway coupled to RNA polyadenylation and cleavage (13). In contrast, transcription of CUTs, some SUTs and other types of ncRNAs (such as small nuclear RNAs) is terminated by a pathway that depends on the RNA-binding proteins Nrd1 and Nab3 (14). These proteins bind to specific sequences in the nascent transcript, interact with RNA polymerase II to terminate transcription and recruit the nuclear exosome for rapid degradation of the transcript (15–20). The binding affinities of Nrd1 and Nab3, and subsequently the termination and degradation efficiency of this pathway, increases with the number of predicted binding sites in a transcript (21,22).

Many CUTs and SUTs originate from nucleosome-free regions (NFRs) at promoters or 3′ ends of protein-coding genes (9). Of these, only a small fraction is transcribed in the same orientation as the mRNA; the majority are transcribed in the antisense orientation with respect to the up- or downstream gene (9). About 48% of SUTs (380 of 794) and 62% of CUTs (465 of 751) overlap with an open reading frame (ORFs) on the other strand; this means that in the absence of introns, most Nrd1 and Nab3-binding site sequences must satisfy the constraints of maintaining a functional protein sequence.

Popularised by the Beatles with their 1966 album *Revolver*, backmasking is used to hide messages in a recording so they can be understood when played in reverse. Backmasking has been an especially controversial topic in the United States with prominent rock bands being accused of hiding satanic messages (a famous case being Led Zepplin's 1982 song *Stairways to Heaven*) leading to anti-backmasking legislation in California (source: Wikipedia). In this paper we explore how the yeast genome encodes overlapping protein-coding and regulatory information using a technique analogous to backmasking.

## MATERIALS AND METHODS

### Annotated transcripts in the *Saccharomyces cerevisiae* genome

The genomic coordinates of 7272 transcripts comprising 5171 ORFs, 847 SUTs and 925 CUTs were obtained from Xu *et al*. (9) (*S. cerevisiae* genome sacCer2, January 2008, from Saccharomyces Genome Database (23)). Transcripts less than 200 bp long were excluded, leaving a total of 7004 transcripts (5164 ORFs, 794 SUTs and 751 CUTs). We identified 440 SUTs and 522 CUTs that overlap with an ORF transcript in the antisense direction by at least 100 bp. Finally, we classified ORFs according to whether they overlap with (i) a stable antisense transcript (430 ORF_SUT_), (ii) an unstable antisense transcript (470 ORF_CUT_) or (iii) have no antisense transcript (4229 ORF_CLEAR_). A total of 95 ORFs with an antisense overlap of less than 100 bp were included in the ORF_CLEAR_ class and 35 ORF overlapping with both SUTs and CUTs by at least 100 bp were excluded. For the PAR-CLIP analysis, amino-acid composition and codon-usage analysis, we excluded ORFs whose sequences (as annotated in sacCer2) did not begin with a start codon and those that overlapped with each other by more than 10 bp, leaving 5092 ORFs (4198 ORF_CLEAR_, 468 ORF_CUT_, 426 ORF_SUT_).

### Predicting Nrd1 and Nab3 binding site sequences

We used the consensus motifs UGUA, GUAG and UGUAG for Nrd1 and UCUU, CUUG and UCUUG for Nab3 to predict binding site sequences in the yeast genome on both strands. Since the 4 bp motifs are subsets of the 5 bp motifs, we always searched for the longest possible predicted binding site sequence. Partially overlapping binding site sequences were counted as two separate sites.

### PAR-CLIP data analysis

We downloaded the Nrd1 and Nab3 PAR-CLIP (Photoactivatable-Ribonucleoside-Enhanced Crosslinking and Immunoprecipitation) data sets (24) from the Gene Expression Omnibus (25) database (GSM791766, Nrd1 Stratalinker Crosslinking and GSM791767, Nab3 Stratalinker Crosslinking) in FASTQ file format using the SRA-toolkit (26). We used the Fastx-toolkit (27) to remove adapter sequences, trim the 3′ ends of reads to 30 nt, remove low quality reads (keeping only sequences where 80% of bases have a score of at least 20) and collapse duplicate reads. Reads were mapped (allowing up to one mismatch) to the 2008 *Saccharomyces cerevisiae* genome sacCer2 using BOWTIE (28) (with command line options bowtie -a -v 1 -m 1). Only uniquely mapping reads were kept. The outputted SAM alignment files were processed with SAMtools (29) for subsequent analysis in R/Bioconductor (30). To quantify protein occupancy ±400 bp of the start and stop codons of ORFs, we binned the mid-points of reads in 10 bp windows.

In the absence of accompanying expression data from the same experiment, we used the tiling array data from Xu *et al*. (9) (downloaded from: *http://steinmetzlab.embl.de/NFRsharing/*) to correct for the expression levels of bound transcripts. The expression data from the three replicates of the deletion mutant of RRP6 were averaged for each 5 bp window in the genome. The sense and antisense expression level for an ORF was computed as the average of the expression data of overlapping positions with the sense and antisense strand, respectively. We normalised the number of PAR-CLIP reads in each bin by the expression level in the sense and antisense for each ORF.

Finally, we calculated the average expression-normalised PAR-CLIP occupancy by dividing the sum of counts in each bin by the total number of ORFs long enough to contribute to the bin. Read counts in intergenic regions were normalised similarly by expression but without taking the strand into account.

### Codon usage analysis in ORFs

We identified adjacent amino acid pairs and triplets with codon combinations that contain an Nrd1 or Nab3 motif in sense or antisense. The 4 bp and 5 bp motifs can be encoded by two amino acids: out of 440 pairs (400 amino acid pairs and 40 pairs of an amino acid and one of the stop codons) 71 have codons containing motifs in sense, 82 in antisense and 10 in both directions. The 5 bp motifs are encoded by three adjacent amino acids if the middle residue is leucine or valine (sense direction), or lysine or tyrosine (antisense direction); these amount to 4 × 440 = 1760 possible codon triplets of which 150 encode a motif in sense and 210 in antisense.

We computed the actual usage of codon combinations for amino acid pairs and triplets for ORF_CLEAR_, ORF_SUT_ and ORF_CUT_. To compute the expected number of binding site sequences in an ORF, we identified all amino acid pairs and triplets that are able to encode an Nrd1 or Nab3 motif, and then calculated the likely number of binding sites sequences in sense and antisense given the underlying codon usage (i.e. the proportion of codon combinations with a motif to those without a motif).

We compared the observed and expected numbers of predicted binding site sequences by computing the residuals for each ORF. Since the residuals for ORF_CLEAR_, ORF_SUT_ and ORF_CUT_ approximate normal distributions, we could assume that they correspond to random errors and that the average codon usages accurately model the predicted binding site occurrences.

We found that the estimates of the numbers of predicted binding sites with single codon usages fit the data less well (with respect to the sum-of-squares-errors of the residuals) and we therefore discarded this approach.

We calculated the codon adaptation index of each ORF with the seqinr R package (31) that uses the relative synonymous codon usage values published by Sharp and Li (32).

## RESULTS

### Predicted Nrd1 and Nab3-binding sites occur at different frequencies in ORFs, CUTs and SUTs

First we assessed the occurrence of the consensus binding motifs for Nrd1 (UGUA, GAUG, UGUAG) and Nab3 (UCUU, CUUG, UCUUG) within the different types of transcripts produced from the yeast genome. Our analyses are based on the annotation of 7004 transcripts by Xu *et al*. (9) comprising 5164 ORF transcripts (including the 5′ and 3′ untranslated regions, UTRs), 751 CUTs and 794 SUTs.

**Figure 1.**
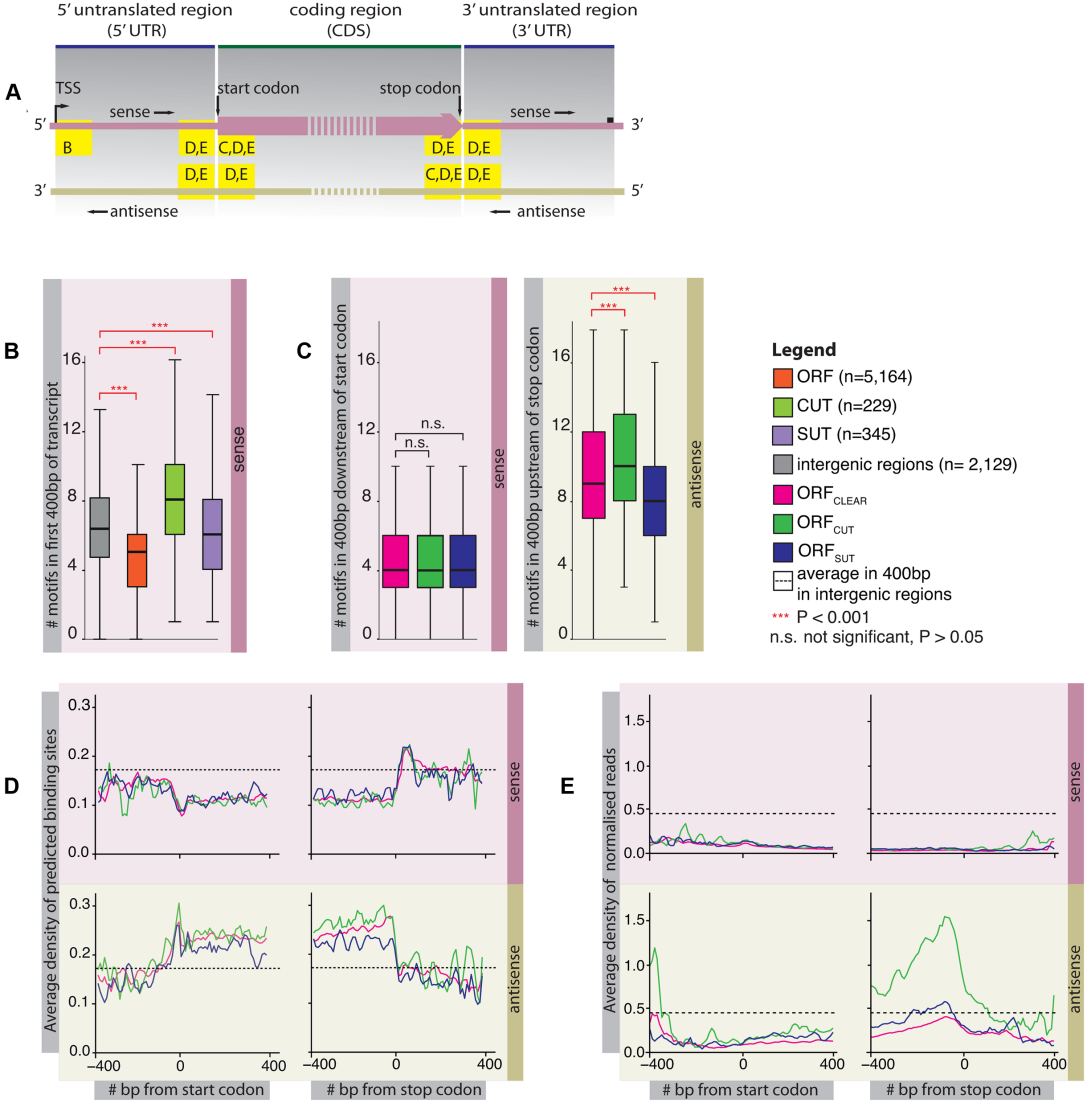
Frequency of Nrd1 and Nab3 binding site sequences in different transcripts. (**A**) Schematic showing regions of interest inside ORF transcripts in the sense and antisense directions for panels B to E. (**B**) Boxplot showing the numbers of predicted binding sites 400 bp downstream of the transcription start site (TSS) in the sense direction of ORF transcripts, CUTs and SUTs. The number of predicted binding sites in 400 bp intergenic regions is shown as a control. The distributions of the numbers of predicted binding sites for different transcript types were compared using the two-sided Wilcoxon rank sum test. All comparisons between the transcript types and the control were statistically significant (alpha = 0.001) and are indicated in red. CUTs contain the most predicted binding sites, followed by SUTs and ORF transcripts. (**C**) Boxplot showing the numbers of predicted binding sites in ORF transcripts 400 bp downstream of the start codon in sense and 400 bp upstream of the stop codon in the antisense direction. ORFs are separated into those with no overlapping transcript (ORF_CLEAR_, n = 4229), those overlapping with a CUT in antisense (ORF_CUT_, n = 470) and a SUT in antisense (ORF_SUT_, n = 430). The distributions of the numbers of predicted sites for different transcript types were compared using the two-sided Wilcoxon rank sum test. All statistically significant comparisons are indicated in red (alpha = 0.001), the others in black. Occurrences of predicted binding sites are almost identical in the sense direction, but differ greatly in antisense depending on the overlapping transcript type. (**D**) Average densities of predicted binding sites in the sense and antisense directions ±400 bp of the start and stop codons of ORFs. Numbers of predicted sites were binned in 10 bp windows and normalised by the total number of transcripts that are long enough to contribute to the bin. As a control for each the 400 bp regions, we show the mean of the average densities of predicted sites in 400 bp in intergenic regions (n = 2129). Densities differ between ORF types only in the antisense direction inside coding regions, with ORF_CUT_ (n = 470) displaying the highest densities, followed by ORF_CLEAR_ (n = 4229) and ORF_SUT_ (n = 430). (**E**) Average densities of PAR-CLIP reads ±400 bp of the start and stop codons of ORF_CLEAR_ (n = 4198), ORF_CUT_ (n = 468) and ORF_SUT_ (n = 426) (excluded are ORFs whose sequences did not begin with a start codon and those that overlapped with each other by more than 10 bp). Reads were binned in 10 bp windows; the number of reads in each bin was normalised by the sense and antisense expression level of the transcript. We further normalised average expression-normalised PAR-CLIP occupancy in each bin by dividing by the total number of transcripts long enough to contribute to the bin. As a control for each of the 400 bp-long regions, we show the average of the binned average number of reads (normalised for expression) in 400 bp in intergenic regions (n = 2129). The differences in the occurrences of predicted binding sites are reflected in differences in protein binding.

The Nrd1-Nab3 complex interacts with the Serine-5 phosphorylated form of RNA polymerase II, which is most prevalent shortly after transcriptional initiation (33–35). Results showing the binding of Nrd1 and Nab3 to increase with the numbers of predicted binding sites (21,22) imply that a larger number of these near the transcription start site suggests a higher degradation rate for transcripts produced from these genes. (See Figure 1A for a schematic indicating these regions.) Figure 1B displays the average frequencies of predicted Nrd1 and Nab3-binding sites in the first 400 bp of transcripts; as previously described (10,17), CUTs encode the largest number of predicted binding sites, reflecting their fast degradation, followed by SUTs and ORF transcripts. In fact, ORF transcripts have far fewer predicted sites than intergenic regions, suggesting that they are avoided in protein-coding sequences.

### Most CUTs and SUTs originate from bidirectional promoters and overlap an ORF in the antisense

Most SUTs and CUTs originate from the 5′ or 3′ NFRs of a gene: the majority are produced from bidirectional promoters (10,36) and are transcribed in the antisense orientation with respect to the gene (9). This means that transcripts originating from a 5′ NFR may overlap with a tandem upstream gene in antisense. 55% of non-overlapping adjacent protein-coding gene pairs are arranged in tandem (n = 2783 of 5105) and 27% are divergently oriented (n = 1387 of 5105); therefore we expect CUTs and SUTs to be transcribed antisense to an ORF. Indeed, we found that most SUTs (449 of 794) and CUTs (522 of 751) overlap with an ORF in the antisense direction with many of them (380 SUTs and 465 CUTs) extending into the coding region. The majority of these overlaps (337 of 449 overlapping SUTs and 430 of 522 overlapping CUTs) are located towards the 3′ end of the ORF transcript.

### ORFs with overlapping antisense SUTs encode fewer predicted antisense Nrd1 and Nab3 binding sites

To explore how the genome encodes distinct transcripts in overlapping genomic regions, but also maintain different binding site frequencies, we compared the number of predicted binding sites encoded in different types of ORFs. We grouped ORFs by the type of the antisense ncRNA: (i) ORF transcripts with an overlapping antisense SUT (ORF_SUT_, n = 430); (ii) those with an overlapping antisense CUT (ORF_CUT_, n = 470); (iii) those with no overlapping transcripts (ORF_CLEAR_, n = 4229).

In the sense direction, as shown in Figure 1C, all three ORF types contain similarly low numbers of predicted binding sites in the first 400 bp of the coding regions. In antisense however, the three ORF types display different frequencies of predicted binding sites: ORF_CLEAR_ contain on average 9.45, ORF_CUT_ 10.05 and ORF_SUT_ 8.6 binding sites in the last 400 bp of the coding regions.

ORF_CUT_ and ORF_CLEAR_ have increased numbers of predicted sites in the last 400 bp of the coding region compared with the first 400 bp (*P* < 10^−4^; two-sided Wilcoxon rank sum test). This suggests that binding sites are encoded in such a way as to degrade any accidental antisense transcripts at the 3′ end of coding genes. Most striking in Figure 1C however are the dramatic differences in the occurrences of predicted antisense sites depending on the overlapping transcript type: ORF_CUT_ have greatly elevated counts compared with ORF_CLEAR_, in line with observations that CUTs are rapidly degraded, while ORF_SUT_ show lower counts than ORF_CLEAR_. We found that the tandem or divergent arrangement of neighbouring ORFs has no effect on predicted binding site frequencies.

### Differences in predicted binding site frequencies are most apparent in coding regions compared with UTRs

We investigated further where predicted binding sites occur within protein-coding genes.

Surprisingly, differences are visible only in the coding regions of ORFs. Figure 1D shows the relative frequencies of binding sites 400 bp up- and downstream of the start and stop codons of ORFs. In the sense direction, the frequencies of predicted binding sites drop dramatically upon transition from the 5′ UTR to the coding region; these low frequencies are maintained until the end of the coding region, at which point they rise again in the 3′ UTR. This is mirrored in the antisense direction, where binding site frequencies are high between the start and stop codons, and low in the 5′ and 3′ UTRs. The difference between different segments of ORFs is highlighted by the fact that 67% of all predicted binding sites in coding regions are on the antisense strand (n = 545 636). In contrast, the predicted binding sites are more evenly distributed in the UTRs with 50% (n = 50 231) on the antisense strand. This suggests that the encoding of predicted binding sites is inherent of the genetic code. This is also supported by the fact that in all ORF types the binding sites are similarly distributed in the sense direction.

However, we observe different frequencies of predicted antisense binding sites between ORF_CLEAR_, ORF_CUT_ and ORF_SUT_ within the coding region in Figure 1D: ORF_CUT_ have high frequencies and ORF_SUT_ have low frequencies of predicted binding sites in antisense compared with ORF_CLEAR_; the differences are most prominent at the 3′ end of ORFs.

### PAR-CLIP data show increased binding to antisense transcripts of ORF_CUT_

In order to assess whether the frequency of predicted binding sites is reflected in the level of protein-binding, we examined the published PAR-CLIP data for Nrd1 and Nab3 (24). Figure 1E shows that protein occupancies detected by PAR-CLIP is reflected in the patterns observed for the predicted binding sites: there is more antisense occupancy at the 3′ end of ORF_CUT_ compared with ORF_CLEAR_ and ORF_SUT_.

### All ORF types code for amino acid sequences that can encode predicted antisense binding site sequences

In the coding regions, codons encoding for particular amino acid sequences constrain the nucleotide sequence and the presence of antisense binding site sequences increases the complexity of the information in these regions. In contrast, UTR sequences are less constrained and antisense binding sites would be easier to encode. It is therefore surprising that the differences in binding site frequencies occur predominantly in the coding regions of genes rather than the UTRs.

**Figure 2.**
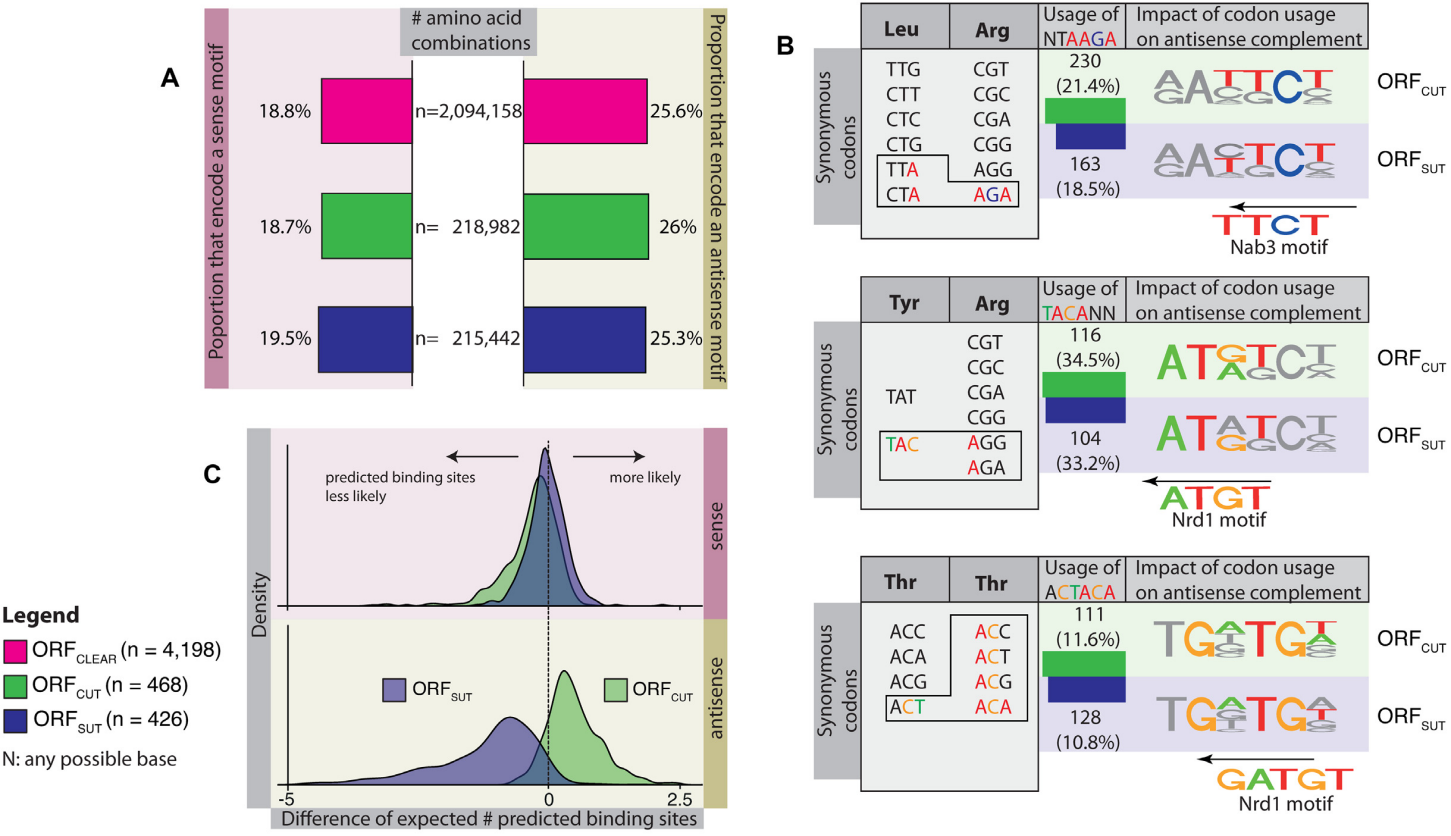
Comparison of amino acid and codon usage in different ORF types. (**A**) Bar plots showing the proportions of amino-acid pairs and triplets that encode Nrd1 or Nab3 motifs in the sense and antisense directions. There is no difference in amino-acid pair and triplet content in the different ORF types. (**B**) Examples of variable codon usage for amino acid pairs in different ORF types. Bar plots show the use of codon combinations in ORF_SUT_ and ORF_CUT_ that encode a binding motif. Although amino acid content is the same across all ORF types, there is a small difference in codon pair usage that affects the occurrence of predicted antisense binding sites. ORF_CUT_ tend to use more codon pairs encoding a motif than ORF_SUT_. (**C**) Distributions of the expected numbers of predicted binding sites in ORF_CUT_ and ORF_SUT_ given the underlying codon usage normalised by the expected numbers of predicted sites given the background codon usage for ORF_CLEAR_. Positive values indicate a larger number of predicted sites than expected (given the background usage), and vice versa for negative values. The distributions for ORF_SUT_ and ORF_CUT_ in the sense direction are centred on 0 (mean = −0.28 and s.d. = 0.5 for ORF_CUT_; mean = 0.00 and s.d. = 0.39 for ORF_SUT_), indicating similar numbers of sites as ORF_CLEAR_. The antisense distribution shows that ORF_SUT_ favour a codon usage pattern that yields fewer predicted binding sites than ORF_CLEAR_ or ORF_CUT_ (mean = 0.48 and s.d. = 0.56 for ORF_CUT_; and mean = −1.15 and s.d. = 1.07 for ORF_SUT_).

To investigate how Nrd3 and Nab1 binding sites can co-exist with protein-coding sequences, we investigated the impact of codon usage across the three ORF types. Figure 2A shows the fraction of amino acid pairs and triplets that could encode a motif in sense or antisense. The proportions are almost equal for the three ORF types, indicating that they all encode for proteins with similar amino acid contents. Therefore, the observed differences in frequencies of predicted antisense sites do not arise from differences in the protein sequence.

### ORF_CUT_, ORF_SUT_ and ORF_CLEAR_ employ different codon usages

In yeast, codon usage is strongly correlated with gene expression to enable efficient transcription and translation (32,37); therefore we examined whether the occurrence of binding site sequences are simply by-products of this codon bias. We calculated a codon adaptation index (CAI) for each ORF. There is a correlation between a high CAI (top 10%, n = 5092) and the number of encoded predicted binding sites in antisense (but not in sense). However, ORF_CUT_ are not overrepresented among ORFs with high CAI: only 22 (4%) are ORF_CUT_ (expected 40% given the overall distribution). The medians of the CAIs are almost equal for the ORF types (0.137, 0.138 and 0.142 for ORF_SUT_, ORF_CUT_ and ORF_CLEAR_, respectively) and are less than half of the median of the ORFs with high CAI (0.357). In summary, the CAI does not explain the enrichment of antisense motifs in ORF_CUT_ and there is no biologically meaningful difference between the distributions of the CAIs for the ORF types.

To investigate the possibility that the differences depend instead on ORF type specific codon usages, we computed the occurrence of adjacent codons containing Nrd1 or Nab3 motifs. The examples in Figure 2B illustrate differences in codon usage across ORF types. For a leucine-arginine pair, ORF_CUT_ prefer a leucine codon with an antisense motif compared with ORF_SUT_ (TTA-AGA or CTA-AGA versus any other combination); for a tyrosine-arginine pair, ORF_CUT_ favour tyrosine codons with an antisense motif (TAC-AGA or TAC-AGG versus any other combination); and for a threonine-threonine pair, ORF_CUT_ favour a threonine codon with a 5 bp antisense motif (ACT-ACA versus any other combination). The last example also shows how the codon choices change for the same amino acid depending on its position in the amino acid pair.

We quantified the extent to which differences in the underlying codon usages across the three ORF types account for the occurrence of predicted binding sites. First we calculated the average usage of codon pairs for each ORF type. Next for every ORF, we calculated the expected numbers of binding site sequences given the underlying codon usage of the corresponding ORF type; the expected numbers resemble the observed distribution of predicted binding site sequences, demonstrating that the average codon usages model the data well (*P* < 10^−4^; Anderson–Darling test for normality on the differences between expected and observed values, data not shown). Finally to compare the impact of the differences in codon usages, we calculated the expected numbers of predicted binding sites in ORF_SUT_ and ORF_CUT_ using the codon usage of the ORF_CLEAR_ class as a control. For each ORF, the difference between the two expected values quantifies the impact of the different codon usages on the occurrence of predicted binding sites: positive values indicate that binding sites are more likely to occur compared with ORF_CLEAR_ and vice-versa for negative values.

Figure 2C displays the distributions of differences for predicted sense and antisense binding site sequences in ORF_SUT_ and ORF_CUT_. In sense, both distributions centre on zero and display only small variances (mean = −0.28 and standard deviation (s.d.) = 0.5 for ORF_CUT_; mean = 0.00 and s.d. = 0.39 for ORF_SUT_); this means that the pairwise codon usages of ORF_SUT_ and ORF_CUT_ do not substantially affect the frequency of predicted binding sites. In contrast, the distributions in the antisense direction are strikingly different: the distribution for ORF_SUT_ stretches to the left (mean = −1.15 and s.d. = 1.07) whereas that for ORF_CUT_ extends to the right (mean = 0.48 and s.d. = 0.56). This demonstrates that the codon usage patterns in ORF_SUT_ and ORF_CUT_ differ from each other and from the background codon usage of ORF_CLEAR_ and enable them to encode different numbers of predicted antisense binding site sequences while maintaining similar amino acid compositions.

## DISCUSSION

Pervasive genome transcription is widespread in the yeast genome and the resulting non-functional ncRNA synthesis must be suppressed. The Nrd1-Nab3 pathway serves as a transcriptome surveillance mechanism to terminate selectively and degrade most aberrant transcription (10), but some ncRNAs escape the rapid degradation and exhibit a function. Here, we have examined how yeast directs this pathway to degrade only certain types of ncRNA by adjusting the number of Nrd1 and Nab3 binding sites.

The ability of the genetic code to encode additional information in parallel to the amino acid sequence of proteins is almost optimal amongst possible genetic codes (38), but it is not known to what extend genomes exploit this potential. In compact genomes, such as in yeast, information is often densely packed within the coding regions of the DNA and the same genomic sequence might be required to encode diverse signals and to avoid interfering with other at the same time. The enrichment of predicted binding site sequences for Nrd1 and Nab3 in the rapidly degraded ncRNAs is an example for these different layers of information: by being enriched or depleted in predicted antisense binding sites, coding sequences encode the relative degradation frequency of RNA produced from its antisense strand and the amino acid sequence in parallel. These genes seem specifically to have evolved sequences that encode only few or many binding sites in the antisense direction. This is possible because of the degeneracy of the codons: whether or not a coding sequence allows stable antisense transcription is controlled by its distinct use of synonymous codons for the same amino acids to either encode or avoid binding site sequences in the antisense direction. Yeast genes seem to have invented backmasking long before it became fashionable in the music industry and uses it to compress information as its own version of MP3.

## CONCLUSION

The Nrd1-Nab3 pathway in yeast rapidly degrades RNA that distinguishes itself from other RNA by the presence of many predicted binding site sequences, while other RNA that have fewer of these are preserved. However, many of these transcripts are encoded in overlapping segments along the genome. We reconciled these facts by demonstrating that coding regions of ORFs not only encode different frequencies of predicted binding sites in sense and antisense directions, but that the stability of antisense RNA is linked to differences in predicted binding site frequencies apparent only in the antisense. We found that this gives rise to a grouping of ORFs where one group prefers different codons that allow them to encode different frequencies of predicted antisense binding sites compared to other ORFs while maintaining the same frequencies in the sense direction. Our results show that yeast uses a parallel genetic code similar to the recording technique backmasking to encode protein coding genes in one direction and the stability of antisense RNA in the reverse of the same stretch of DNA.
